# COGNITIVE FRAILTY: PREVALENCE, RISK FACTORS, AND THE RISK OF MORTALITY IN CHINESE MIDDLE-AGED AND OLDER ADULTS

**DOI:** 10.1093/geroni/igad104.3721

**Published:** 2023-12-21

**Authors:** Hongting Ning, Hui Feng, Junxin LI

**Affiliations:** Central South University, Changsha, Hunan, China (People’s Republic); Central South University, Changsha, Hunan, China (People’s Republic); Johns Hopkins University, Baltimore, Maryland, United States

## Abstract

To examine the age-specific prevalence, risk factors, and magnitude of the association between cognitive frailty and all-cause mortality in Chinese middle-aged and older adults. We used baseline (2011) and follow-up waves (2013, 2015, and 2018) data from the China Health and Retirement Longitudinal Study (CHARLS). The age group was categorized as middle-aged, old, and very old. Factors associated with cognitive frailty and the associations between cognitive frailty statuses and mortality were examined using multinomial logistic regression and Cox proportional hazards models, respectively. Totally 7947 participants were included in this study, and the overall prevalence of cognitive frailty was 12% (middle-aged, 11%; old, 10%; very old, 26%; P< 0.0001). Cognitive frailty was independently associated with rural residential status, long sleep duration, lack of social participation, depressive symptoms, and multimorbidity among all the age groups. Over the 7-year follow-up, participants with cognitive frailty were at a higher risk for all-cause mortality than the robust group (HR 1.70, 95%CI 1.12-2.57 for the middle-aged group, HR 1.70, 95%CI 1.02-2.84 for the old group, and HR 2.06, 95%CI 1.17-3.65 for the very old group). Pre-cognitive frailty was associated with a higher mortality risk in the middle-aged group only (HR 1.34, 95%CI 1.01-1.79). The prevalence of cognitive frailty in middle-aged adults was comparable with that in the old group. Cognitive frailty, a significant risk factor for mortality, was associated with multiple factors in middle-aged and older adults, calling for integrated cognitive frailty prevention strategies. Noticeably, pre-cognitive frailty in middle-aged people increases mortality risk and requires special attention.

